# Obesity and leptin in breast cancer angiogenesis

**DOI:** 10.3389/fendo.2024.1465727

**Published:** 2024-10-08

**Authors:** Courtney B. Lagarde, Kapil Thapa, Nicole M. Cullen, Mackenzie L. Hawes, Khudeja Salim, Megan C. Benz, Sophie R. Dietrich, Brandon E. Burow, Bruce A. Bunnell, Elizabeth C. Martin, Bridgette M. Collins-Burow, Ronald M. Lynch, Van T. Hoang, Matthew E. Burow, Jennifer S. Fang

**Affiliations:** ^1^ Department of Medicine, Section of Hematology and Oncology, Tulane University School of Medicine, New Orleans, LA, United States; ^2^ Tulane University Cancer Center, New Orleans, LA, United States; ^3^ Department of Cell and Molecular Biology, Tulane University School of Science and Engineering, New Orleans, LA, United States; ^4^ United States Department of Agriculture Southern Regional Research Center, New Orleans, LA, United States; ^5^ Department of Microbiology, Immunology, and Genetics, University of North Texas Health Science Center, Fort Worth, TX, United States; ^6^ Department of Physiology, College of Medicine, University of Arizona, Tucson, AZ, United States; ^7^ Department of Pharmacology, Tulane University School of Medicine, New Orleans, LA, United States; ^8^ Department of Surgery, Tulane University School of Medicine, New Orleans, LA, United States; ^9^ Department of Physiology, Tulane University School of Medicine, New Orleans, LA, United States

**Keywords:** leptin, angiogenesis, breast cancer, obesity, hyperleptinemia, tumor microenvironment

## Abstract

At the time of breast cancer diagnosis, most patients meet the diagnostic criteria to be classified as obese or overweight. This can significantly impact patient outcome: breast cancer patients with obesity (body mass index > 30) have a poorer prognosis compared to patients with a lean BMI. Obesity is associated with hyperleptinemia, and leptin is a well-established driver of metastasis in breast cancer. However, the effect of hyperleptinemia on angiogenesis in breast cancer is less well-known. Angiogenesis is an important process in breast cancer because it is essential for tumor growth beyond 1mm^3^ in size as well as cancer cell circulation and metastasis. This review investigates the role of leptin in regulating angiogenesis, specifically within the context of breast cancer and the associated tumor microenvironment in obese patients.

## Introduction

All cells, including cancer cells, depend on the vasculature for oxygen, nutrient access and waste removal to support their basic metabolic functions. This is particularly true for fast-growing solid tumors, where the densely packed and metabolically active tumor stroma quickly drives the tumor microenvironment to become acidic and hypoxic. As hypothesized by Dr. Judah Folkman in 1971, cancer must at some point during tumor progression become dependent on the blood vasculature; and, in the absence of tumor neovascularization, the tumor core quickly becomes necrotic, constraining solid tumors to <2mm^3^ in size due to the limitation imposed by oxygen diffusion distance (7). Many cancers escape this constraint by inducing nearby blood vessels to undergo angiogenesis, such that new blood vessels sprout towards and invade the tumor mass. Resulting blood flow into the tumor brings critical oxygen and nutrients that can support further tumor growth and expansion (8). In addition to being a critical step in tumor growth, cancer neovascularization also provides an essential conduit for metastasis – the process by which some cancer cells escape the primary tumor and invade the blood (or lymphatic) circulation to seed a secondary tumor at a distal tissue site.

Regulation of the angiogenic signaling axis is affected by several disease states, including chronic obesity, which drives a systemic pro-inflammatory state that promotes angiogenesis (9, 10). As obesity becomes more and more prevalent, it is increasingly important to understand how obesity contributes to the pathology of many diseases, including cancer. There is a strong correlation between poor outcomes in cancer and obesity: overweight and obese patients are at increased risk of cancer mortality (11). Additionally, obesity is associated with higher incidence of many cancers, including cancer affecting colorectal, endometrial, and breast tissue (12, 13).

Obesity is associated with higher risk of developing breast cancer – especially in post-menopausal women – and is also associated with larger tumors, positive lymph node status, and shorter disease-free interval with decreased survival (1–3, 14, 15). Furthermore, most breast cancer patients are obese or overweight at diagnosis (1). Thus, there is a pressing need to better understand how obesity interacts synergistically with breast cancer to affect patient outcomes. In particular, identifying the molecular targets which contribute to the obesity-breast cancer axis may allow for the development of novel interventions for breast cancer patients that specifically address obesity’s contribution to their disease progression. In this review, we focus on the role of the adipokine leptin, whose blood concentration is greatly elevated in proportion to patient obesity.

Leptin is a peptide hormone produced and secreted by adipocytes and adipose stem cells (ASCs) and is the primary regulator of long-term balance between energy storage and expenditure (4). Leptin also appears to be an upstream regulator in the obesity-breast cancer axis (5). Obesity-induced hyperleptinemia contributes to poor outcomes across breast cancer subtypes, with increased metastases in triple negative breast cancer (TNBC) and increased tumor growth in estrogen receptor positive breast cancer (5, 16, 17). Separately, leptin is well-established as a potent inducer of angiogenesis, in both physiologic and pathologic contexts (10, 18) pointing to a logical link between elevated leptin and enhanced tumorigenesis. Given the critical role that angiogenesis plays in cancer development and patient survival outcomes, the role of leptin in driving angiogenesis in obese breast cancer patients is of critical relevance.

## Angiogenic dysregulation in obesity

**Figure 1:**
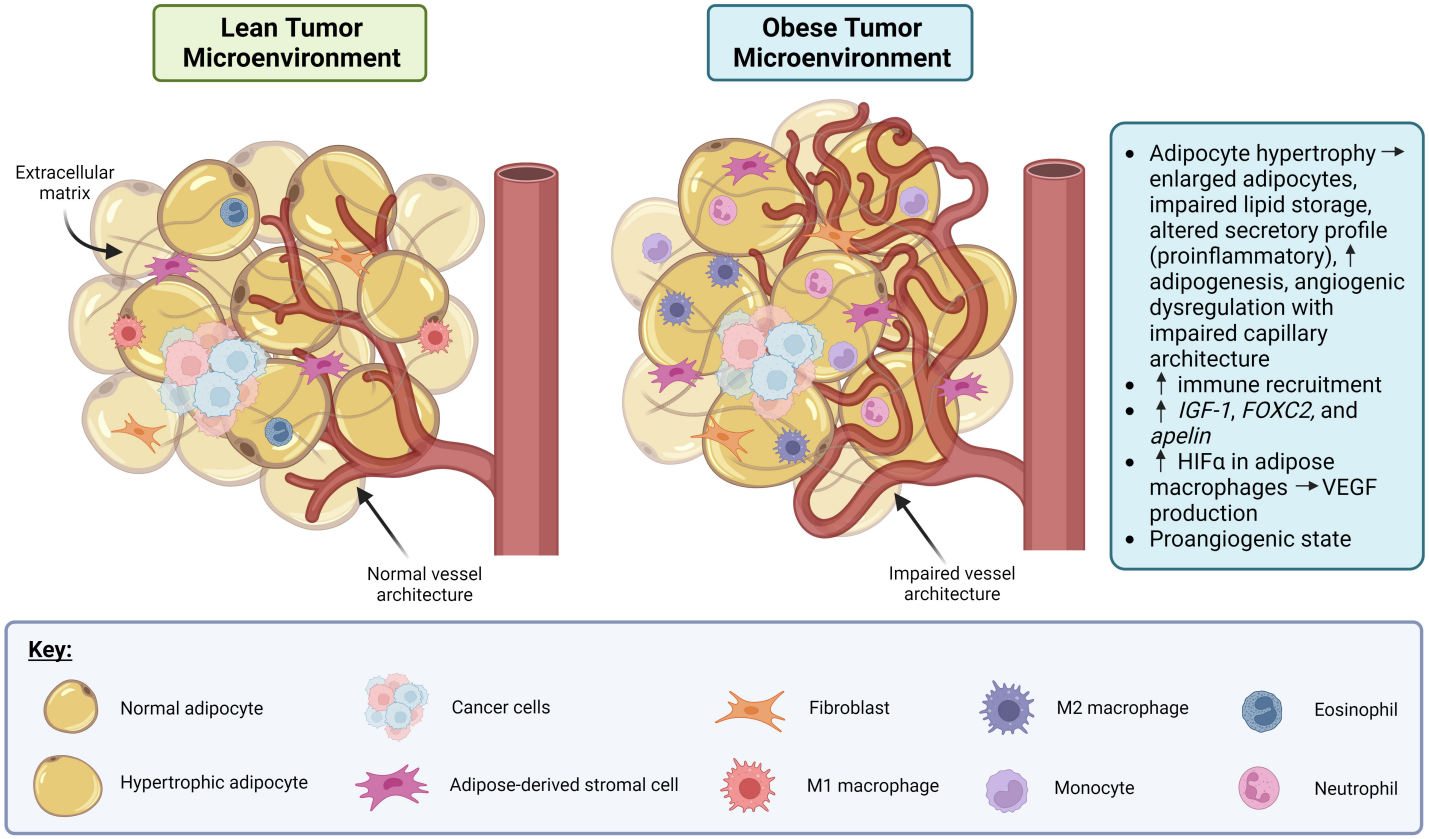
Obesity promotes dysregulation of angiogenesis and induces a pro-inflammatory state in adipose tissue in the context of cancer. In the lean tumor microenvironment (TME), the adipocytes, vasculature and immune involvement are all normal. In the obese TME, adipocyte hypertrophy can occur resulting in the adipocytes becoming enlarged, lipid storage impairment, a pro-inflammatory secretory profile, and angiogenic dysregulation. Also, in the obese TME, immune recruitment is increased via production of IGF-1, FOXC2, and apelin. Macrophages in obese adipose tissue display increased HIFα which drives production of VEGF, leading to a pro-angiogenic state. Created in Biorender. Benz, M. (2024).

The link between obesity and angiogenic dysregulation is well-established in both cancer and non-cancer (e.g., diabetes and cardiovascular disease) contexts, which are more common in obese individuals (19). **Figure 1** depicts the effect of obesity-associated dysregulation of angiogenesis in the context of cancer. Adipose tissue is highly vascularized, and several metabolic signaling axes that affect adipose tissue function also regulate angiogenesis. For example, genes that govern adipose tissue expansion in obesity also affect angiogenic signaling, including insulin-like growth factor 1 (*IGF-1)*, which is produced by adipocytes near capillaries and which stimulates endothelial cell proliferation and angiogenesis (20). Genes associated with angiogenic patterning, including forkhead box C2 (*FOXC2)* and apelin (APLN), are also expressed in adipocytes in the context of obesity, which may contribute to adipocyte metabolic function and insulin sensitivity (20). High caloric intake and the resulting obesity-related adipocyte hypertrophy are associated with impaired capillary architecture of associated adipose tissue as demonstrated in **Figure 1** (20). Interestingly, many signaling pathways elevated in obesity are also present in cancer and appear to target angiogenic signaling (21). For example, *HIF-1α* is elevated in adipose tissue macrophages with obesity, and its expression decreases after weight loss (22). *HIF-1α* is also expressed in many cancers, including breast cancer (23), where it drives the production of the pro-angiogenic signal VEGF. Circulating VEGF itself is also elevated in both obese individuals (9, 24) and in some cancers (25), including breast cancer, where it appears to be prognostic for patient outcome and survival (26, 27). Together, these data suggest that angiogenic dysregulation serves as a nexus between obesity and breast cancer, such that obesity might promote a pro-angiogenic state in the tumor microenvironment that is favorable for breast cancer progression and metastasis.

## Angiogenesis in cancer

Endothelial cells (ECs) form the vascular endothelium, a continuous single-cell layer that faces the lumen of blood vessels and separates circulating blood from surrounding tissues (8, 28). In addition, ECs play critical cell signaling roles in controlling tissue-level blood flow and regulating immune cell adhesion and extravasation (8). Lastly, ECs drive new blood vessel sprouting during angiogenesis, in which avascular tissue secretes pro-angiogenic signals (e.g., VEGF) that stimulate nearby ECs. ECs respond to this pro-angiogenic signal by expressing matrix metalloproteases (MMPs) that digest the extracellular matrix, allowing them to partially detach from the vascular endothelium, proliferate, and migrate towards the source of the pro-angiogenic signal to establish a new vessel sprout (29). Angiogenesis is a critical step during embryonic development to initially establish blood vasculature, and many pro-angiogenic signals are frequently reactivated postnatally both under physiological conditions (e.g., female reproductive tissue cycling, wound healing, etc.) and in pathological settings (e.g., cancer) (30, 31).

Angiogenic activation of ECs is determined by the relative balance of pro- (e.g., VEGF, ANG-1, FGF, etc.) (29) vs. anti- (e.g., thrombospondin-1, angiostatin, interferon α/β) angiogenic factors, a phenomenon referred to as the “angiogenic switch”. Dysregulation of this switch is a classic hallmark of cancer (6, 32). The earliest evidence of this was the observation that implantation of tumor cells induced neovascularization (33). In another classic study, Folkman isolated a soluble factor from human tumor cells -- called Tumor-Angiogenesis Factor (TAF) -- that rapidly stimulated endothelial cells to form capillaries (7). It has since been established that many cancers overexpress several soluble pro-angiogenic signals, as well as their upstream regulators (e.g., Ras, Myc, etc.) (32). Beyond inducing sprouting angiogenesis, tumor cells can also access vascular blood flow by inducing pathological vasculogenesis (the formation of vessels *de novo* by recruiting circulating endothelial progenitor cells) (34), or by migrating towards and “co-opting” native healthy vessels (35). Indeed, an immunohistochemical study of a human inflammatory breast cancer (IBC) xenograft model revealed that pathological vasculogenesis may be a significant contributor to breast cancer neovascularization (36). Lastly, some tumor cells can transdifferentiate directly into EC and integrate into healthy vasculature, a process termed “vasculogenic mimicry” (37). These non-angiogenic mechanisms may also be affected by obesity in breast cancer but are beyond the scope of the current review.

## Angiogenesis in breast cancer

As solid tumors develop, central regions distant from the existing vasculature become hypoxic leading to the stabilization and accumulation of the transactivating factor Hypoxia Inducible Factor-1α (HIF-1α). Like most solid tumors, breast cancer is highly vascularized due to elevated production of soluble pro-angiogenic signals, such as VEGF and FGF that are downstream of HIF-1α in precursor lesions and early-stage cancer (38, 39). HIF-1α induces VEGFA production by both breast cancer stroma and tumor-associated macrophages, which make up ~50% of the total cellular mass in breast cancers (40). White adipose tissue of surrounding breast tissue, which produces a variety of factors that directly regulate the breast cancer tumor microenvironment, also contribute to pro-angiogenic signaling by releasing VEGF, basic fibroblast growth factor (bFGF), matrix metalloproteases (MMPs), and interleukin 8 (IL-8) (41, 42). Excess HIF-1α-induced VEGF stimulates local vessel growth and can even enter the systemic blood circulation where it is critical for establishing vasculature within the developing breast cancer. Consistent with these observations, VEGF production and tumor angiogenesis are significantly disrupted in the absence of elevated HIF-1α (43). Indeed, high circulating serum VEGF levels are prognostic for breast cancer progression and patient survival (26, 27, 44), likely because it correlates to tumor vascularity and metastatic potential.

In addition to the previously described data linking circulating VEGF levels to patient outcomes in breast cancer (26, 27, 44), recent transcriptomic studies have directly linked increased expression of many pro-angiogenic genes with poor patient outcomes (44, 45). For example, Guarischi-Sousa et al. conducted a 1000-patient cohort study in which they identified a 153 gene signature for pathological angiogenesis that was predictive for poor prognosis and decreased patient survival in luminal A, luminal B, and basal, but surprisingly, not HER2-positive, breast cancer subtypes (46, 47). This finding is consistent with clinical trials showing that anti-VEGF therapy is not effective in HER2-positive breast cancer patients (AVEREL Trial) (46, 47). Interestingly, some genes identified in the study by Guarischi-Sousa et al. are regulated by obesity-associated leptin signaling, including *SERPINE1* and *VEGFA* (16, 48).

## Anti-angiogenic therapy in breast cancer

Because of the essential role that tumor neovascularization plays in tumor progression, anti-angiogenic therapy offers, at least in theory, a strategy for halting both tumor growth and metastatic invasion. In reality, however, the angiogenic switch is regulated by a complex (and often functionally redundant) nexus of pro- and anti-angiogenic signals. This may explain why anti-angiogenic monotherapies – such as a humanized monoclonal antibody that binds to and sequesters VEGF-A rendering it signaling-incapable (Avastin/Bevacizumab) has produced only limited improvements for breast cancer patients (49). Anti-angiogenic therapies and their current use in breast cancer is summarized in **Table 1**. Despite several clinical studies documenting Bevacizumab treatment (either alone or in combination with conventional chemotherapy) to be initially effective in breast cancer (50–52), the RIBBON-1 study found that this did not ultimately translate into significantly increased overall survival (52). This finding, along with the observation that Bevacizumab increases toxicity of standard chemotherapy in this trial, resulted in the FDA withdrawing approval for Bevacizumab for combination treatment of metastatic Her2/neu negative breast cancer in 2011 (53). Although it remains unclear why Her2-negative breast cancer is resistant to Bevacizumab when combined with other chemotherapies, one possibility is that overexpression of other pro-angiogenic/pro-lymphangiogenic growth factors in this cancer type may compensate for inhibition of VEGF-A. Alternatively, ordinarily leaky and disorganized blood vessels may “normalize” following anti-angiogenic therapy which increases access of cytotoxic drugs to the tumor stroma, thereby enhancing their efficacy (54, 55). Indeed, anti-angiogenic drugs have been shown to prune the tumor vasculature by eliminating excess ECs, leading to reduced tumor vessel density in mice (56, 57) while improving tumor vascular perfusion (58). Whether this effect is less likely to occur in some breast cancer types -- thereby contributing to the apparently reduced efficacy of anti-angiogenics in some breast cancers -- is unknown.

**Table 1:** Anti-Angiogenic drugs and clinical trials in breast cancer.

Drug	Target/Mechanism of Action	Current use in Breast Cancer	FDA-labelled indications	Non FDA-labelled indications	Relevant or Ongoing Clinical Trials	Sources
Bevacizumab (Avastin)	Humanized Anti-VEGF monoclonal antibody	FDA approval withdrawn in 2011, but currently can be used off-label to treat metastatic HER2 negative breast cancer, in combination with paclitaxel or other chemotherapy	Cervical cancer, Glioblastoma multiforme, Liver Carcinoma, colorectal cancer, Renal cell carcinoma, Nonsquamous non-small cell lung cancer, ovarian cancer	Metastatic HER2 negative breast cancer. Macular degeneration, other retinopathies	RIBBON-1, NCT05192798 NCT04739670	(64, 65)
Ramucirumab (Cyramza)	Monoclonal antibody targeting VEGFR-2	Not currently used in breast cancer, trials did not demonstrate improvement in Overall Survival (OS) or Progression Free Survival (PFS)	Esophogastric cancer, gastric cancer, liver carcinoma, colorectal cancer, non-small cell lung cancer	Urothelial carcinoma	NCT01234402	(64, 66)
Sorafenib (Nexavar)	Tyrosine kinase inhibitor targeting VEGFR, PDGFR, and RAF	Not currently used in breast cancer, trials did not demonstrate improvement in OS or PFS	Liver carcinoma, renal cell carcinoma, thyroid cancer	Acute myeloid leukemia, gastrointestinal stromal tumor	NCT00544167 NCT00096434	(64, 67)
Sunitinib (Stutent)	Tyrosine kinase inhibitor targeting VEGFR1, VEGFR2, fetal liver tyrosine kinase receptor 3, c-KIT, PDGFRa, and PDGFRb	Not currently used in breast cancer, trials did not demonstrate improvement in OS or PFS	Gastrointestinal stromal tumor, pancreatic neuroendocrine tumors, renal cell carcinoma	Thyroid cancer	NCT00471276 NCT00246571	(64, 68)
Vandetinib (Caprelsa)	Tyrosine kinase inhibitor of VEGFR2, VEGFR3, EGFR and RET	Not currently used in breast cancer, trials did not demonstrate improvement in OS or PFS	Medullary thyroid carcinoma	None	NCT02530411	(64, 69)
Axitinib (Inlyta)	2nd generation pan-VEGFR Tyrosine kinase Inhibitor	Not currently used in breast cancer, trials did not demonstrate improvement in OS or PFS	Renal cell carcinoma	Metastatic renal cell carcinoma	NCT00076024 NCT05904730	(64, 70)
Pazopanib (Votrient)	Tyrosine kinase inhibitor targeting VEGFR, PDGFR, FGFR, and c-KIT	Not currently used in breast cancer, trials did not demonstrate improvement in OS or PFS	Renal cell carcinoma, soft tissue sarcoma	Gastrointestinal stromal tumor, Thyroid cancer	NCT01466972 NCT00558103	(64, 71)

In addition to these possibilities, obesity may be a confounding factor in treating breast cancer with an anti-angiogenic approach using Bevacizumab. The expression of inflammatory cytokines and angiogenic factors like interleukin-6 (IL-6) and fibroblast growth factor 2 (FGF2), which contribute to resistance against anti-angiogenic therapies (59–61), are upregulated in adipocyte-rich hypoxic regions (62). Those regions are more prevalent in tumors from obese mice which likely contributes to anti-VEGF (anti-mouse VEGF antibody-B20-4.1.1) therapy resistance (63). The same study also showed increased systemic concentration of IL-6 and/or FGF-2 in obese patients suggesting possible activation of alternative angiogenic pathways in association with obesity (63).

Nonetheless, combination anti-angiogenic/cytotoxic chemotherapy approaches (including some strategies involving Bevacizumab) have been more promising in a spectrum of solid tumor malignancies, including renal cell, pancreatic, gastric, head and neck, colorectal, glioblastoma, hepatocellular, cervical, ovarian, and importantly several types of breast cancer (64); among these are some (e.g., pancreatic, ovarian, etc.) that – like breast cancer – are more prevalent with patient obesity. Thus, while anti-angiogenics remain a viable strategy for cancer treatment, a better understanding of how angiogenic signaling is dysregulated by both obesity and tumor stroma is needed to better understand how current and new anti-angiogenic approaches can be adapted to improve efficacy in obese breast cancer patients.

## ASC-derived leptin is a key regulator of the breast microenvironment

The breast environment is a complex milieu comprised of multiple cell types including adipocytes, ductal epithelial cells, vascular endothelial and mural cells, fibroblasts, immune cells and adipose-derived stromal/stem cells (ASCs). ASCs are a mesenchymal cell type that play key roles in energy and lipid homeostasis, and serve as a major source of endocrine signals, including leptin, which positions ASCs as crucial regulators of signaling crosstalk between the distinct cell populations within healthy (and diseased) breast tissue (28, 75, 76). In particular, ASC contribution to the leptin signaling axis in breast stroma is critical for healthy adipose function in the breast. ASCs are altered by obesity and metabolic diseases, including insulin resistance, dyslipidemia, atherosclerosis, hypertension, and certain cancers (72). In obese patients, for example, increased fat mass dysregulates inflammation- and angiogenesis-associated genes in association with reduced adipose tissue oxygenation, likely contributing to altered adipose tissue homeostasis (73). Adipocyte hypertrophy and hyperplasia also alter the secretory profile of ASCs, resulting in excess production of cytokines and adipokines, including leptin (74), which promote inflammation in obesity (72). For these reasons, ASCs are key regulators of obesity-associated sequelae and disease due in part to their control of leptin signaling.

Adipocyte- and ASC-derived leptin (28, 75, 76) regulates energy balance, metabolism, endocrine signal regulation and immunity (77–80), and increased circulating leptin levels are associated with obesity and its associated sequelae. Indeed, leptin is considered a master regulator of obesity: high levels (i.e., hyperleptinemia) have been shown to significantly increase the risk of glucose and lipid metabolism-associated disorders in obese patients (81), and increased leptin is both necessary and sufficient to induce hypertension in obese mouse models (82).

Hyperleptinemia is also associated with reduced survival rates in obesity-related cancers, including breast cancer (83). This may be due to several effects of altered leptin signaling, including increased cancer cell resistance to anti-estrogenic drugs (e.g., Tamoxifen) (78, 84). Dysregulated leptin signaling in breast cancer may influence tumor growth by acting both directly and indirectly on tumor neovascularization and angiogenesis. For example, in a model of 4T1 mammary carcinoma cells grown adjacent to the mammary fat pad of syngeneic mice (85), leptin receptor antagonists reduced tumor growth by up to 90% and this was associated with reduced VEGF and VEGFR tumor expression. Similarly, leptin knockout in obesity-derived adipocytes reduced tumor growth of triple negative breast cancer (BT20) which was associated with reduced expression of angiogenesis-promoting genes (e.g., *SERPINE1*, *SNAI2*, *IL-6*, *TWIST1*, and *PTGS2*) (86). Leptin has also been found to drive metastasis in both an ER+ and ER- context, with leptin knockout in obese ASCs resulting in reduced motility of BC cell lines *in vitro* and reduced metastasis in an *in vivo* patient-derived xenograft model of TNBC (5, 87). Thus, in addition to its other regulatory roles, leptin plays a central role in tumor angiogenesis and metastasis in breast cancer (5).

## Leptin regulates angiogenesis and metastasis in breast cancer

**Figure 2:**
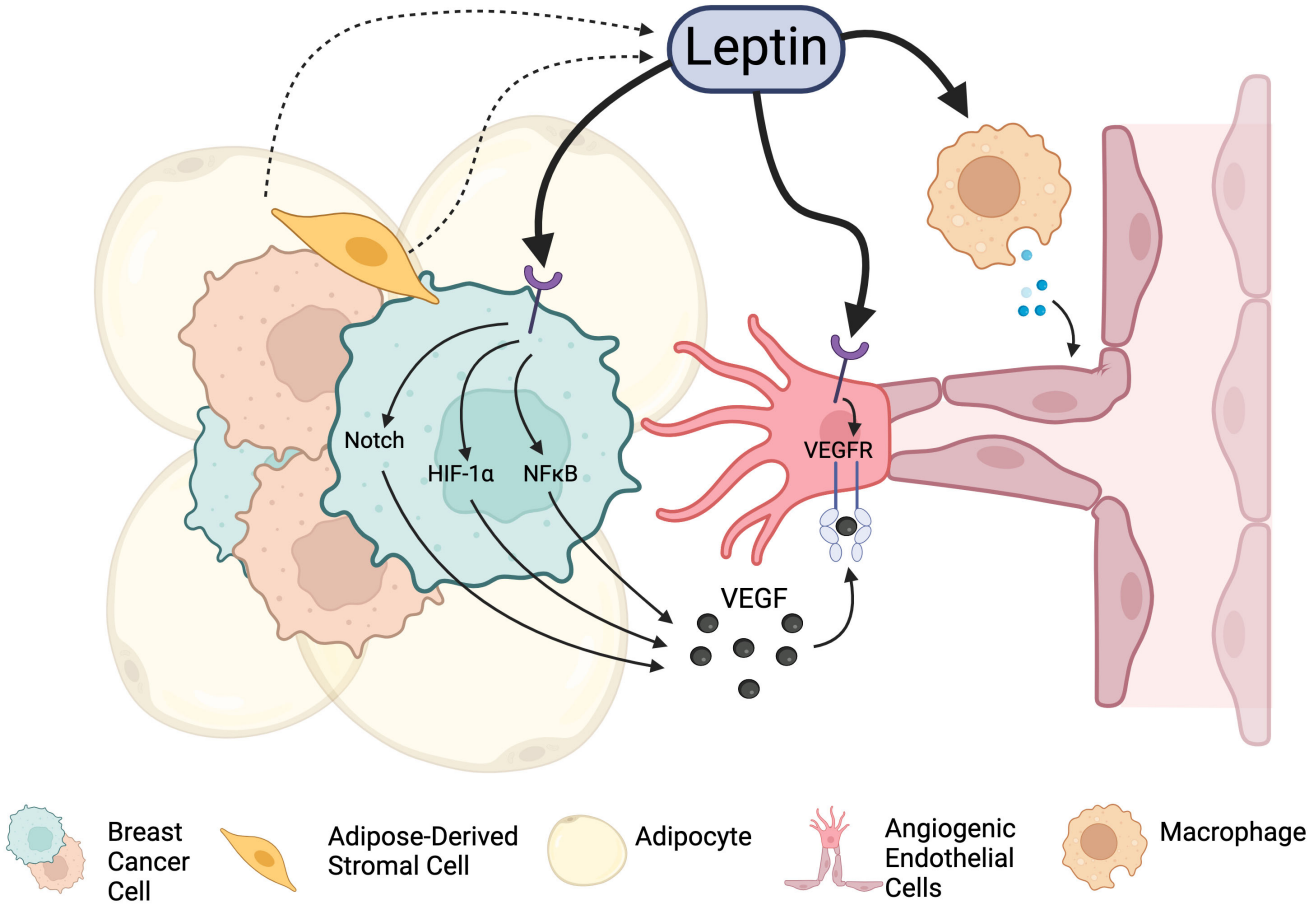
Leptin signaling promotes angiogenesis in the breast cancer TME through integrations with Leptin receptors. Leptin acts directly on cancer cells to stimulate the release of angiogenesis-promoting factors. It also can act on endothelial cells directly to promote proliferation and vascular expansion. Leptin can also indirectly promote angiogenesis through other cell types and factors present in the tumor microenvironment, including immune cells and alteration of estrogen signaling. Created in Biorender. Fang, J.S. (2024).

Leptin is a well-known inducer of angiogenesis, and exogenous leptin classically promotes angiogenic sprouting in corneal explant models (88). In breast cancer, leptin promotes angiogenesis via several mechanisms (**Figure 2**): i) leptin acts *directly* on breast cancer stroma to promote the production of soluble pro-angiogenic signals (e.g., VEGF (89), FGF); ii) leptin acts *directly* on ECs of tumor blood vessels to promote angiogenic activation and enhance angiogenic responses (90); and iii) leptin *indirectly* promotes angiogenesis by priming the tumor microenvironment for neovascularization through effects on estrogen signaling and immune cell activation. Each of these mechanisms will be explored more in depth in the subsequent sections.

In addition to these pro-angiogenic effects, leptin also regulates adipocyte capillary fenestration (91), resulting in vessels that may become more permeable to cancer cell extravasation and metastasis. This is a critical effect of leptin on tumor blood vessels in adipose tissue but is beyond the scope of the present review.

## Leptin activates breast cancer-expressed leptin receptors to promote angiogenic signaling

Leptin signals directly to breast cancer stromal cells by paracrine and/or autocrine signaling activation of cell surface leptin receptors (LEPRs), ObR-a and ObR-b. Both receptor isoforms are strongly expressed in human breast cancer cell lines and are significantly overexpressed in human breast cancer in comparison to non-transformed mammary gland tissue (92, 93). In addition to promoting tumor cell proliferation, leptin signaling activation upregulates cancer cell production and secretion of soluble VEGFA via NFκB, Sp1, and HIF-1α signaling to induce angiogenic sprouting into the growing tumor mass (85, 94). In particular, HIF-1α binds directly to the hypoxia response element (HRE) of the *VEGFA* promoter in mammary tumor cells (95), and when the *HRE* site was deleted from the *VEGFA* promoter in a luciferase reporter assay, leptin-induced luciferase signal was significantly reduced (86). Gonzalez-Perez and colleagues also reported that leptin-mediated production of HIF-1α and NFκB involves different signaling pathways: the former via canonical (JAK2/STAT3, PI3K/AKT1, and MAPK/ERK1/2) and the latter non-canonical (p38, JNK and to a lesser extent PKC) downstream signaling pathways (86).

Leptin also regulates pro-angiogenic VEGF/VEGFR2 via a distinct Notch/IL-1 signaling axis termed Notch, IL-1, Leptin crosstalk outcome (NILCO) (96). Leptin upregulates Notch signaling effector expression (including Notch1/4 receptors, and Notch ligands Jag1 and Dll4) which upregulates Notch target genes (*HEY2*, *Survivin*) and enhances IL-1 expression. Notch signaling is required for leptin-induced pro-angiogenic activation (96), and inhibition of IL-1R type 1 blocks leptin-induced Notch signaling activation, as well as downstream upregulation of HEY2, Survivin, and VEGF/VEGFR2.

Taken together, the above-described studies underscore leptin’s critical role in regulating the expression of soluble pro-angiogenic VEGF in breast cancer cells via multiple intermediate signaling pathways. Thus, overexpressed VEGF in breast cancer stroma critically supports tumor expansion by activating nearby blood vessels to undergo sprouting angiogenesis and tumor neovascularization.

## Leptin activates endothelial-expressed LEPRs to directly promote angiogenic activation of tumor ECs

In addition to directly regulating breast cancer cells to increase VEGF expression in the tumor microenvironment, leptin can potently activate angiogenesis by acting directly on tumor endothelial cells. First, Sierra-Honigmann and colleagues found that vein endothelial cells (VECs) express LEPRs *in vitro*, and that leptin can induce angiogenesis in normal but not leptin receptor-deficient rat corneas (88). Leptin may activate angiogenesis in multiple ways, including by increasing endothelial cell expression of pro-angiogenic factors such as VEGFRs and MMPs, including MMP2 and MMP9, both classic hallmarks of pro-angiogenic activation of ECs (97–99). Leptin also can enhance the activation of proliferation- and migration-associated PI3K/Akt and MAPK signaling pathways (97–99). As a result, ECs display a dose-related angiogenic response to leptin that promotes EC survival, proliferation, migration, and growth (98, 100), as well as their organization into capillary-like structures (100, 101). Direct leptin stimulation of ECs is critical for the angiogenesis that underlies healthy adipose tissue extension (100), as well as for angiogenesis and tumor neovascularization in cancers such as glioblastoma *in vitro* (101).

Leptin activation of endothelial-expressed leptin receptors promotes VEGF signaling by directly trans-phosphorylating the VEGFR2 receptor and increasing Notch expression which in turn increases VEGF/VEGFR expression in EC (102). VEGFR and Notch are necessary for leptin-induced direct effects on EC: a study by Lanier et al. showed that leptin-induced proliferation and EC tube formation were impaired by either VEGFR or Notch signaling inhibition. The study further demonstrates that leptin-induced VEGFR-1 and VEGFR-2 transphosphorylation was necessary for leptin’s actions (102). Separately, Garonna et al. showed that VEGFR2 activation also increases endothelial cyclooxygenase 2 (COX-2) expression to induce angiogenesis, and that this response is enhanced in a p38^MAPK^- and PI3K/Akt -dependent manner (98). COX-2 inhibitors, such as celecoxib, reduce tumor growth in breast cancers (103), indicating the need for further study to determine whether inhibition of these downstream components affects leptin-induced angiogenesis in a breast cancer context.

Additionally, leptin regulates the expression of endothelial nitric oxide synthase (eNOS), which produces vasodilatory nitric oxide (NO) to regulate local vasomotor responses and control downstream blood flow and tissue perfusion (100, 104). This is especially relevant in the context of both cancer and obesity-associated fat expansion, as both can lead to metabolic imbalance and excessive production of reactive oxygen species (ROS). Resulting oxidative stress can in turn drive endothelial dysfunction due to reduced NO bioavailability (104), and also can be compensated for by leptin-mediated angiogenesis to re-establish tissue (or tumor) blood flow and support metabolic waste removal.

Lastly, leptin may also regulate angiogenesis through the Wnt signaling pathway. Wnt is a potent pro-angiogenic signal, and *Wnt2* deficiency is lethal in 50% of embryos due to placental and embryonic vascular defects (105). ECs express several types of Wnt ligand, Wnt receptor, and downstream β-catenin-associated transcription factors (106). Using an *in vitro* Matrigel assay, Wnt/β-catenin signaling activation is sufficient to promote the formation of capillary networks (106), possibly by transcriptional regulation of pro-angiogenic VEGF (107), Interleukin 8 (IL8) (108), and MMPs (106). Interestingly, some studies have implicated leptin as an upstream regulator of Wnt signaling in ECs. Yu et al. found that leptin activates Wnt pathways in ECs *in vitro* and *in vivo*, and that silencing RNA knockdown of Wnt signaling effectively blocks angiogenesis *in vitro* (109). In these studies, Akt knockdown reduced GSK-3β phosphorylation and β-catenin translocation. Thus, β-catenin translocation can be triggered by either the Wnt/Frizzled or Leptin/Akt signaling axes, and these pathways appear to converge at the level of GSK-3β. Separately, leptin was found to regulate breast cancer cell growth in a Wnt/β-Catenin-mediated manner (110). This same study also showed upregulation of leptin in serum of breast cancer patients in comparison to the healthy individuals. However, it remains unclear to what extent leptin/Wnt signaling crosstalk supports breast cancer growth by specifically promoting tumor neovascularization, or if other outcomes of leptin/Wnt signaling activation predominate in breast cancer tissue.

## Indirect leptin-mediated mechanisms of pro-angiogenic activation

**Figure 3:**
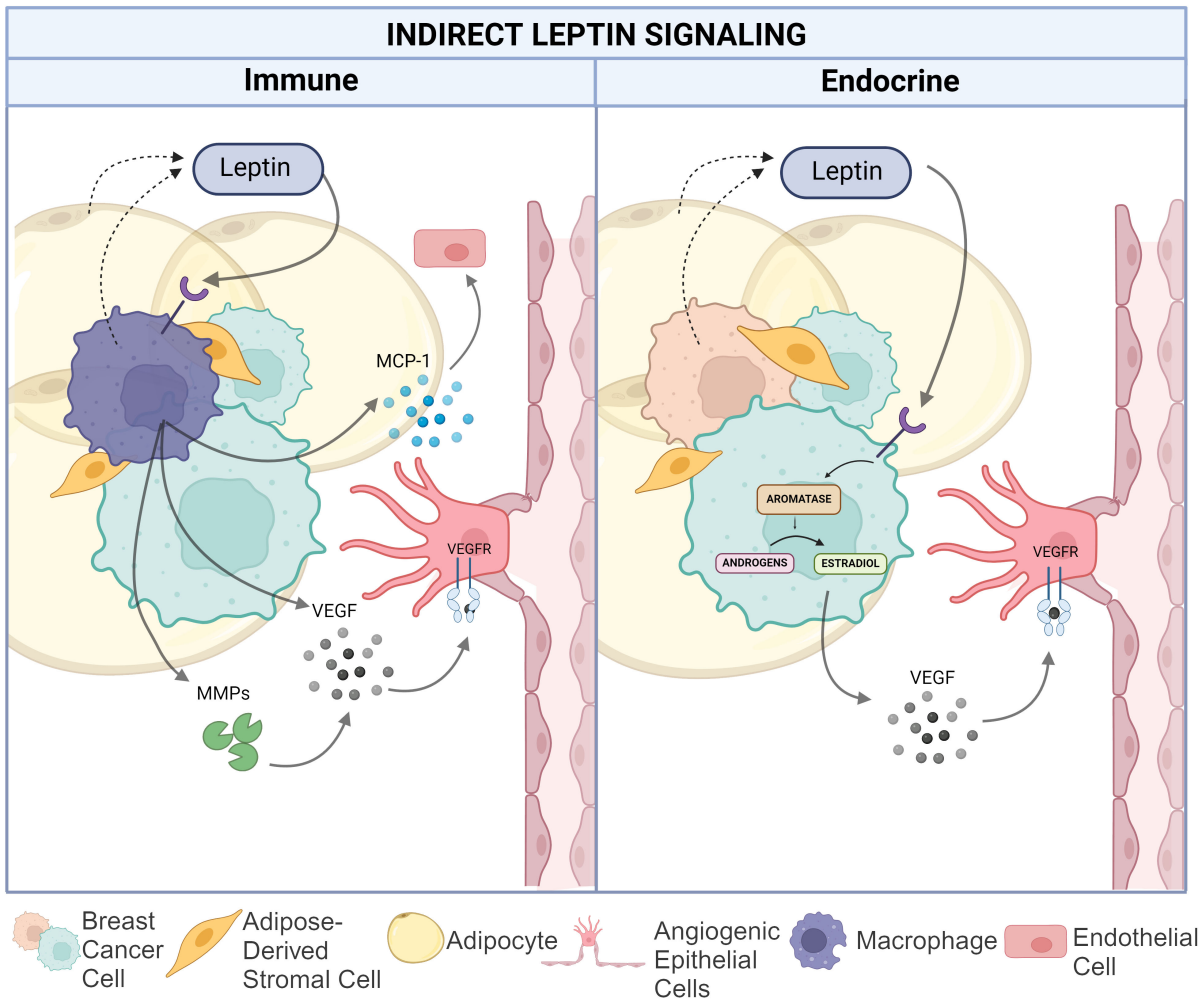
Indirect leptin-mediated pro-angiogenic action in the breast cancer TME. The left panel depicts the impact of leptin on macrophage-mediated angiogenesis via increased matrix metalloproteinase and VEGF production as well as MCP-1 recruitment of endothelial cells. The right panel illustrates leptin induced conversion of androgens to estradiol in TNBC cells resulting in increased VEGF production. Created in Biorender. Hawes, M. (2024).

Beyond leptin signaling onto breast cancer and endothelial cells to directly control the VEGF/VEFR signaling axis and other pro-angiogenic pathways, leptin can also induce a pro-angiogenic TME through estrogen-signaling in breast adipocytes and by promoting pro-inflammatory signaling by tissue- (or tumor-) resident macrophages, as shown in **Figure 3**. Both may potentially contribute to angiogenesis in the setting of breast cancer.

### Leptin-regulated estrogen signaling in adipocytes and breast cancer cells

Estrogen and the estrogen receptor (ER) are essential drivers and therapeutic targets for hormone receptor-positive breast cancer. Leptin interacts with estrogen pathways through several mechanisms to indirectly promote angiogenic signaling in cancer cells and in the tumor microenvironment. In ER+ breast cancer cells, leptin increases aromatase expression and enzymatic activity, leading to an increase in overall estrogen levels (111). In post-menopausal women, circulating estrogen is low, but it can still remain high in breast adipose tissue thereby serving as an estrogen source for breast tumors (112). Multiple mechanisms for maintaining post-menopausal estrogen production in breast have been proposed, including suppression of LKB1/AMPK, suppression of the MAPK/p53 axis, and upregulation of COX2 (83). Leptin-induced aromatase expression also occurs in adipose stromal cells within the tumor microenvironment (113).

Although the link between leptin and estrogen signaling has not been explicitly studied in the context of angiogenesis, estrogen signaling is known to strongly regulate vessel growth. Treatment of cardiac microvascular ECs with estrogen (E2), for example, reduces oxidative stress and inflammatory cytokines, and also promotes endothelial progenitor cell recruitment to sites of vascular damage (114, 115). In breast cancer, increased E2 bioavailability also promotes angiogenesis. In ovariectomized mice, treatment with E2 improved endothelial cell maturation, stabilization, and organization, as well as increased expression of VEGF-A, VEGFR-3, and VEGFR-1 (116). Another study found that Matrigel plugs mixed with ER+ breast cancer cells from estrogen-treated, ovariectomized mice had lower expression levels of soluble VEGFR-1, increased bioavailability of VEGF protein, and increased angiogenesis, compared to untreated controls (117). Increases in *VEGF* mRNA and VEGF protein expression with estrogen treatment also have been observed *in vitro* with ER+ cell line, MCF7 (118, 119). Lastly, estrogen signaling can induce HIF1-α nuclear translocation to enhance the release of soluble VEGF (120).

### Leptin-regulated cytokine production and immune responses

Leptin also regulates immune responses – both locally in the breast as well as systemically – in a manner that modulates angiogenic responses. Leptin becomes elevated during infection and inflammation, and it regulates cytokine production and immune cell responses in patients (121, 122); both strongly influence angiogenesis. Structurally related to pro-inflammatory cytokines (e.g., IL-6, IL-11, IL-12, and Oncostatin M), leptin is upregulated by cytokine-induced signaling activation, including by tumor necrosis factor alpha (TNF-α) and IL-6 (123). Interestingly, the long form of the LEPR OB-Rb shares structural and functional similarities to IL-6-type cytokine receptors (124).

Leptin acts on multiple cell types of healthy mammary tissue and breast cancer, including on mammary-resident (and tumor-resident) macrophages, which can contribute to tumor neovascularization. Many circulating immune cells including CD34+ hematopoietic cells, CD4+ and CD8+ lymphocytes, and platelets express the long form of the leptin receptor Ob-Rb (124), as well as toll-like receptor 4 (TLR4) and inflammatory chemokine C-C chemokine ligand 2 (CCL2) receptor, that enable them to crosstalk with cancer and adipose tissue (125). Macrophages, on the other hand, specifically express the short form of leptin receptor (124). As a result, leptin is a strong chemoattractant for monocytes even at concentrations as low as 1pg/ml (126). Additionally, the treatment of M2 macrophages with leptin activates the IL-8 promotor through the p38/MAPK and MAPK/ERK1/2 pathways leading to increased IL-8 secretion resulting in enhanced tumor growth *in vivo* and increased migration and invasiveness in breast cancer cell lines (127). Indeed, even slight changes in interstitial leptin concentration can significantly enhance chemoattraction and recruitment of monocytes and macrophages into breast adipose tissue, leading to altered breast cancer migration and metastatic potential in mouse models (126, 128, 129).

Leptin stimulates macrophages to secrete pro-inflammatory and pro-angiogenic cytokines, including IL-1, IL-18, TNFα, NO and pro-angiogenic VEGF (128, 130, 131). Leptin-induced IL-8 is pro-angiogenic in several human cancers, including in colorectal and lung cancer (132), and may also activate angiogenesis in breast cancer. Indeed, IL-8 signaling activation promotes the formation of dense vascular networks in the breast stroma during malignant progression (133). In triple negative xenografts, knockdown of the leptin receptor decreased the level of infiltrating macrophages and CCL2 expression, reducing the secretion of pro-angiogenic cytokines and VEGF (134). Separately, leptin-induced NFκB activation also leads to increased expression of IL-1, which in turn promotes VEGF production in breast cancer *in vitro* (89). This leptin-cytokine-angiogenesis axis is particularly interesting because it has been proposed as a therapeutic target in certain diseases. In a model of obese breast cancer in humans, it has been suggested that targeting leptin regulation of cytokine production could be potentially therapeutic by decreasing angiogenesis and regulating stem cell function in breast tumors (135).

In addition to directly promoting cytokine production, leptin feeds back onto both the innate and adaptive immune systems, contributing to a systemic pro-inflammatory and pro-angiogenic state. Regarding the adaptive immune system, leptin critically regulates circulating T-cell number and function. Mice lacking leptin production (*ob/ob*) or leptin receptor (*db/db*) exhibit thymic atrophy and decreased T-lymphocyte levels, while exogenous leptin rescues thymus proliferation in the thymus of *ob/ob* mice (136) and can promote thymic proliferation and survival in humans (123). In addition to direct effects on T-cell maturation, Leptin upregulates IL-2 and IFN-γ, and downregulates IL-4 levels, which regulate the phenotypic switching between Th1 and Th2 CD4+ T-cells (124, 137). Leptin also suppresses anti-inflammatory regulatory T-cell (Treg), further contributing to a pro-inflammatory state (137). In patients with chronic obstructive pulmonary disease (COPD), for example, increased leptin is associated with decreased Treg cells in the lung due to inhibition of T-cell glycolysis, an essential metabolic pathway for T-cell survival and conversion into Tregs (138). In a separate study of the gastric mucosa, Treg function was affected by *Helicobacter pylori* vaccination in a leptin-dependent manner (139).

In terms of the innate immune system, leptin regulates the function of macrophages in various ways (137), in addition to being a chemoattractant (129). Leptin triggers calcium influx in macrophages to enable the “leading edge” and “ruffling” structures that allow macrophages to migrate towards leptin, but not towards other chemoattractants such as MCP-1 (126, 140), and also modulates macrophage cytokine secretion profiles (130). In a murine model of Roux-en-Y gastric bypass (RYGB), post-bypass mice exhibited decreased leptin levels in association with macrophage polarization toward the M2 phenotype (141) and increased Treg levels (142). This suggests a link between weight loss and leptin signaling in macrophage polarization. In a separate study using a human Salmonella typhimurium infection model, gram-negative bacteria activate LEPR signaling in human macrophages. When the *LEPR* gene was absent, macrophages exhibited increased lysosomal functionality, reducing bacterial burden and inflammation (143). These and other data underscore how leptin functions as a bridge between inflammation and metabolism and can play a central role in the pro-inflammatory signaling that occurs in obesity and other metabolic disorders.

Leptin-induced pro-inflammatory signaling and immune cell activation can indirectly enhance angiogenic responses in breast cancer and other tissues through several mechanisms. These include inducing macrophages to secrete pro-angiogenic VEGF to stimulate angiogenesis, releasing extracellular matrix metalloproteases to degrade local extracellular matrix (144), and producing CCL2 (also known as MCP-1) to recruit endothelial cells and smooth muscle cells for neovascularization (45). M1 vs. M2 macrophages are distinctly involved in different phases of tumor neovascularization, and appropriate temporospatial regulation of M1 and M2 macrophages determines the extent of angiogenic sprouting and vascular growth. Recently, a research group found in a three-dimensional microfluidic angiogenesis model that M2 macrophages are pro-angiogenic in glioblastoma, while M1 macrophages are anti-angiogenic (141). CD4+ T-cells also regulate and promote angiogenesis. Tregs express Neuropilin-1, VEGF, and Leptin (145), and it was recently shown that Tregs stimulate angiogenesis in gliomas through VEGF signaling and in lung ischemia (146, 147), and CD4+ T-cells may regulate angiogenesis via IL-22 signaling (148).

## The potential for leptin-based therapeutics to inhibit aberrant angiogenic processes

Given the role of leptin signaling in promoting and regulating angiogenesis, the potential for leptin-based therapeutics in addressing angiogenic disease and aberrant angiogenic processes is being studied in both cancer and non-cancer contexts. Allo-aca, a small peptide inhibitor of LEPRs, has been found to inhibit VEGF signaling effects in an ophthalmic neoangiogenesis model (149). Coroniti et al. found that crosstalk between leptin and VEGF in retinal and corneal endothelial cells resulted in increased proliferation and motility; Allo-aca treatment inhibited chemotaxis and expression of VEGF-induced pathways *in vitro* as well as choroidal neovascularization *in vivo* (149). Leptin signaling antagonists also appear to improve outcomes in cardiovascular disease: leptin induces thrombotic events in a mouse model of pulmonary embolism, but event frequency is reduced–and overall survival improved–by treatment with a leptin-neutralizing antibody (150). Leptin signaling also has been found to significantly contribute to post-infarction cardiac remodeling and heart failure (151). In rats, treatment with a LEPR-neutralizing antibody was able to prevent post-injury myocardial hypertrophy and significantly improve cardiac function in rats following coronary artery ligation (151). While not directly relevant to angiogenesis in cancer, these studies indicate that pharmacological agents can interrupt the leptin signaling axis in vascular disease.

Many LEPR antagonists exhibit anti-cancer activity in various cancer subtypes and models, though few studies have specifically examined the effect of LEPR inhibitors on tumor angiogenesis (152). In a colorectal cancer xenograft model, LEPR inhibition (via Allo-aca) blocks NILCO-induced expression of VEGFR1/2, although this did not affect the overall tumor growth rate in mice (153). In breast cancer, Gonzalez et al. reported that a LEPR inhibitor (pegylated LEPRA2) significantly reduces endothelial cell proliferation and tube formation by preventing leptin-induced upregulation of VEGFR and subsequent Notch activation (85). LEPRA2 reduced VEGF secretion and expression in 4T1 murine breast cancer cells, and treatment with LEPRA2 significantly reduced VEGF levels in serum, as well as tumor growth in 4T1 implant tumors (85). However, it remains unclear from these studies whether the observed reduction in tumor growth with LEPR inhibition is due solely to effects on angiogenesis, as LEPRA2 also reduces Cyclin D expression in breast cancer cells *in vitro* (85). Nonetheless, the same group later confirmed that LEPR2A also reduces tumor growth rate and VEGF/VEGFR2 levels in ER+ (MCF7) and ER- (MDA-MB-231) breast cancer cells and tumor xenografts (154).

Despite these promising results, ongoing challenges limit the use of LEPR antagonists to inhibit angiogenesis in breast cancer patients. As discussed above, leptin is a well-established immune system modulator (155), and the LEPR antagonist Allo-aca affects T cell differentiation and function in mice (156). While this may be desirable in autoimmune conditions, further research is needed to determine how combining this effect with chemotherapy drugs in cancer might impair therapies that require functional T-cells to be effective, such as PD-1 and PDL-1 inhibitors.

Additionally, because leptin is crucial for satiety and metabolic functions, LEPR antagonism often results in weight gain, an unwanted side effect that may limit patient adherence. Furthermore, some data indicate that weight gain is associated with poor outcomes in BC patients making it unclear whether potential benefits of LEPR inhibition of breast cancer angiogenesis would outweigh the negative effects of associated weight gain (10). However, Zabeau et al. have recently created a LEPR antagonist antibody that uncouples the downstream metabolic and immune functions of LEPR (157). Using a single-domain antibody that selectively inhibits LEPR-EGFR crosstalk, they observed no significant changes in food intake, weight gain, or visceral or subcutaneous fat composition in LEPR antibody-treated mice compared to vehicle controls. However, they also observed that treatment blocked leptin’s protection against starvation-induced splenic and thymic atrophy, leading to a decrease in lymphocytes compared to leptin treated controls (157). Thus, it may be possible to avoid unwanted weight gain with LEPR inhibition, and further research is needed to develop such uncoupled LEPR antagonists for clinical use.

## Discussion

Given the prevalence of obesity in the general population and specifically among breast cancer patients, it is critical to understand how obesity contributes to breast cancer pathology. In this review, we describe how hyperleptinemia in obesity can drive angiogenesis in breast cancer through its actions on both cancer cells themselves and on other cells in the tumor microenvironment, including endothelial cells and immune cells. Leptin is also able to promote angiogenesis indirectly via upregulation of estrogen signaling. This supports the importance of local stromal remodeling in breast cancer: hyperleptinemia can drive estrogen-dependent alterations to the TME, which can have profound effects on tumors regardless of BC hormone receptor status.

Most studies referenced in this review article were performed using patient-derived xenograft or murine models of breast cancer; however, in the future, more studies exploring the difference in intratumoral microvascular density in obese vs. non-obese patients would provide valuable insight into how obesity (and associated hyperleptinemia) affects angiogenic signaling in breast cancer. Nonetheless, while anti-angiogenic therapies are effective in many cancers, their effectiveness is likely modulated by obesity-associated signaling, such as through the leptin signaling axis. Thus, therapies that jointly target leptin signaling alongside anti-VEGF therapy may help breast cancer patients – many (but not all) of whom are obese – in which conventional anti-VEGF combination therapy is less effective, such as in Her2-negative breast cancer. Future studies should further focus on developing such approaches, which would likely prove beneficial to all breast cancer patients and particularly those who present with obesity at the time of breast cancer diagnosis.
